# Co-existence of patent ductus arteriosus and left brachiocephalic artery: a case report

**DOI:** 10.1186/s13019-015-0224-y

**Published:** 2015-02-22

**Authors:** Mange Manyama, Erick Mazyala, William Mahalu

**Affiliations:** 1 Department of Anatomy, Catholic University of Health and Allied Sciences, Mwanza, Tanzania; 2 Department of Surgery, Bugando Medical Centre, Mwanza, Tanzania

## Abstract

Patent ductus arteriosus (PDA) may exist with other cardiovascular anomalies, which must be considered at the time of diagnosis. We report a rare co-existence of PDA and a variant of aortic arch branching pattern in a 12-year old Tanzanian female patient during surgery to close a PDA. In this case, the ‘left brachiocephalic trunk’ was seen to arise from the arch of aorta distal to the origin of the right brachiocephalic trunk. We discuss the relevant literature, its potential embryologic development and clinical significance.

## Background

The patent ductus arteriosus (PDA) is one of the more common congenital abnormalities of the great vessels in which a persistent vascular structure connects the proximal descending aorta to the roof of the main pulmonary artery. The incidence of PDA is estimated to be as high as 1 in 500 [1]. PDA results from failure of closure of the fetal ductus arteriosus after birth. The fetal ductus arteriosus which normally closes spontaneously after birth is an important structure that is essential for normal fetal development. Its function during fetal period is to permit right ventricular output to be diverted away from the high-resistance pulmonary circulation [1]. Persistence of ductal patency after the first few weeks of life is abnormal as it leads to variable left-to-right shunt of blood from the aorta to the pulmonary artery. Clinical presentation varies from those who are completely asymptomatic to those with severe congestive heart failure, depending on the size of the PDA and the underlying cardiovascular status of the patient [2].

The arch of aorta is a continuation of the ascending aorta, ascending diagonally back and to the left over the anterior surface of the trachea, then back across its left side and finally continues as the descending thoracic aorta. As far as the branches of the aortic arch are concerned, there is a plenty of variations in their pattern of origin. In about 65% of individuals, there is a separate origination of the brachiocephalic trunk, left common carotid and left subclavian arteries springing from the vessel’s convex aspect [3]. In other aortic arch branching pattern, each of the four arteries (brachiocephalic trunk, left common carotid and left and right subclavian arteries) originates independently from the arch of the aorta [3]. In more rare patterns, the right and left brachiocephalic arteries originate from the arch of the aorta or the left common carotid artery originates from the brachiocephalic trunk [3,4].

Anatomically, anomalies of the aortic arch can be classified into two major categories namely, sidedness or position of the aortic arch (the course of the aortic arch itself) and anomalous branching patterns of the aortic arch (order and pattern of branches from the arch of aorta) [5]. Aortic arch branching patterns have been further classified into five main groups including double aortic arch, right aortic arch with mirror-image braching, right aortic arch with abnormal branching, right aortic arch with abnormal branching and cervical aortic arch [5].

Anomalies in branching pattern of the arch of the aorta result from abnormal or incomplete regression of one or more of the embryonic branchial arches. Reports from genetic studies have indicated that deletion of chromosome 22q11 is associated with anomalous branching pattern of the aortic arch [6,7]. Aortic arch anomalies can cause a variety of physiological complications including, Tracheobronchial compression, oesophageal compression and abnormal blood flow patterns which particularly occurs when there is isolation of a subclavian, carotid or innominate artery (i.e. origin of these vessels from the proximal pulmonary artery by means of a ductus arteriousus) [5].

Few cases have been reported in which PDA co-exists with other cardiac anomalies such as congenital aortic valvular diseases as well as Coronary artery fistulas [8].

In this case report, we present a rare co-existence of PDA and a variant of aortic arch branching pattern where the ‘left brachiocephalic trunk’ was seen to arise from the arch of the aorta distal to the origin of the brachiocephalic trunk. We discuss their embryological, clinical and surgical implications.

## Case presentation

The present report describes a finding of a left common carotid and subclavian arteries arising from a ‘left brachiocephalic trunk’ in a 12-year-old Tanzanian girl undergoing closure of a Patent Ductus Arteriosus (PDA) at Bugando Medical Centre in Tanzania. Prior to surgery at the age of three years old, the girl had presented to the hospital complaining of persistent cough and shortness of breath. Physical examination revealed the typical machinery murmur of a PDA. Echocardiography confirmed a PDA which was 10 mm wide with a left to right shunt. In addition, the left side of the heart was enlarged and there was a moderate pulmonary hypertension. Unfortunately the hospital does not have angiographic investigations available.

**Figure 1:**
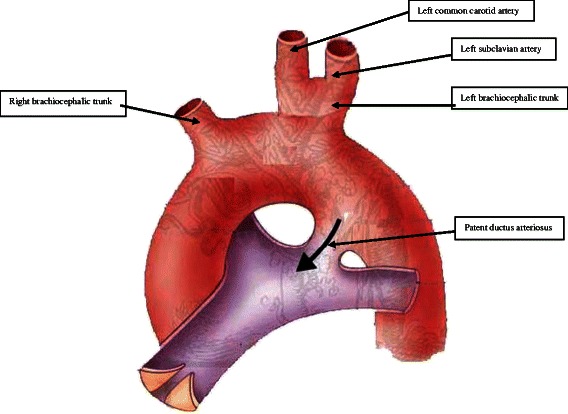
**Schematic diagram shows the patent ductus arteriousus and anomalous branching pattern of the aortic arch.**

Under general anesthesia, a left thoracotomy was done. There was about 100 mls of pleural effusion but the lung was normal. A palpable thrill on the PDA was felt. The pleura and mediastinal fascia were then dissected and reflected medially to expose the arch of aorta, descending aorta and the PDA. The PDA was about 5 mm long and 12 mm wide (Figure 1). It was dissected and double ligated using 5 silk tie. While dissecting the left subclavian artery, it was noticed that the left common carotid and subclavian arteries had a common origin from a ‘left brachiocephalic trunk’ which measured about 10 mm long and 8 mm wide (Figure 1). The arch of the aorta was dissected further proximally to show the origin of the ‘right brachiocephalic trunk’. The diameter of the right brachiocephalic trunk was about 8 mm. The child was discharged on the seventh post-operative day.

## Conclusions

In normal cardiovascular development, the sixth aortic arch gives off an important branch that grows toward the developing lung bud. On both sides, the proximal part of the sixth aortic arch becomes the proximal segment of the right and left pulmonary arteries [9]. The distal segment of this arch on the right side loses its connection with the dorsal aorta and disappears while on the left side the distal portion persists during intrauterine life as the ductus arteriousus [9]. The fetal ductus arteriosus is an important structure which is essential for normal fetal development, allowing most of the blood from the right ventricle to bypass the fetus’s fluid-filled non-functioning lungs. Premature constriction or closure may lead to right heart failure, resulting in fetal hydrops [10]. Upon closure after birth, the ductus arteriosus becomes a fibrous band known as the ligamentum arteriosum. Failure of closure of the ductus arteriousus after birth leads to PDA.

In majority of individuals, the aortic arch gives rise to three classical branches, namely the brachiocephalic trunk, the left common carotid artery and the left subclavian artery. However, various branching pattern of the aortic arch have been described [3,4]. In some cases, the left common carotid artery have been shown to originate from the brachiocephalic trunk [3,4]. Rarely the left common carotid and the left subclavian arteries have been shown to have a common origin in the form of the left brachiocephalic trunk from the aortic arch [3]. Most irregularities of aortic arch branching pattern result from persistence of parts of aortic arches that usually disappear, or from disappearance of parts that normally persists.

The left common carotid artery is formed from the proximal part of the left third aortic arch while the left subclavian artery is derived from the left seventh intersegmental artery [11]. During the fourth week of development, the initial location of the left subclavian artery is shifted as a result of embryo’s cephalic folding, growth of the forebrain and elongation of the neck. The left subclavian artery which is initially fixed in the distal arm bud shifts its point of origin from the aorta at the level on seventh intersegmental artery to an increasingly higher point until it comes close to the origin of the left common carotid artery [9]. The existence of left brachiocephalic artery in this case could not be explained by the existing literature. However, we postulate two scenarios that can explain this finding. Common origin of the left common carotid and left subclavian arteries (through the left brachiocephalic trunk) could suggest that the left subclavian artery is also derived from the proximal part of the left third aortic arch. Alternatively, it could be a result of fusion of the proximal parts of the left common carotid and left subclavian arteries as a result of cranial shift of the left subclavian artery too close to the origin of the left common carotid artery.

In the present case, the clinical presentations were all consistent with the PDA. However, PDA may also exist with other cardiac anomalies, which must be considered at the time of diagnosis. PDA has been found in individuals with congenital aortic valvular diseases, as well as Coronary artery fistulas [4]. It has been reported that in the presence of complex congenital heart defects, the usual anatomy of the ductus may not be present due to the fact that anatomic abnormalities can vary widely but most are common with complex aortic arch anomalies [12]. Structures that have been mistaken for the patent ductus arteriosus (PDA) in surgical procedures include the aorta, the pulmonary artery, and the carotid artery [12]. Although the co-existence of PDA and ‘left brachiocephalic trunk’ in this case did not pose any surgical challenge, knowledge of variations in the branching pattern of the arch of the aorta is of great importance in patients who have to undergo four-vessel angiography, aortic instrumentation, or supraaortic thoracic, head and neck surgery [13]. Knowledge on variation of origin and course of a great vessel arising from the aortic arch is of great clinical value in minimizing complications that may arise especially during surgical procedures in the superior mediastinum and the root of neck. Further, while operating around the arch of aorta and the descending aorta (either for coarctation of aorta, or PDA), it is important to correctly identify the branches of the arch of aorta.

Traditional 2-dimensional imaging techniques, such as radiography, echocardiography, and angiography, have shown to be limited in the diagnosis of extracardiac intrathoracic vascular anomalies [14,15]. The use of multidetector-row computed tomography (MDCT) has been shown to be more superior over the traditional 2-dimensional techniques due to the fact the 3-dimensional MDCT are able to display the anatomy of aortic anomalies (PDA or aortic arch anomalies) and the spatial relationship of adjacent structures [14,15]. In the present case, the left brachiocephalic trunk was not picked by echocardiography. Some of the major Hospitals in Tanzania, including Bugando Medical Centre are in the process or have just established cardiothorax surgical units. Introduction of MDCT at these centres is therefore highly recommended since it will provide valuable information to surgeons prior to surgery and subsequently reduce potential risks that can result from missed diagnosis of cardiovascular anomalies.

## Consent

A written consent was obtained from the patient’s parents for publication of the article. A copy of the written consent is available for review by the Editor-in-Chief of this journal.
